# Multiple Mycotoxin Contamination in Medicinal Plants Frequently Sold in the Free State Province, South Africa Detected Using UPLC-ESI-MS/MS

**DOI:** 10.3390/toxins14100690

**Published:** 2022-10-08

**Authors:** Julius Ndoro, Idah Tichaidza Manduna, Makomborero Nyoni, Olga de Smidt

**Affiliations:** 1 Department of Life Sciences, Faculty of Health and Environmental Sciences, Central University of Technology, Free State, Private Bag X20539, Bloemfontein 9300, South Africa; 2 Centre for Applied Food Sustainability and Biotechnology (CAFSaB), Central University of Technology, Free State, Bloemfontein 9300, South Africa; 3 Research, Development and Innovation Department, National Biotechnology Authority, 21 Princess Drive Newlands, Harare, Zimbabwe

**Keywords:** medicinal plants, mycotoxins, Ultra High Pressure Liquid Chromatography–Tandem Mass Spectrometry, contamination, street vendors, *muthi* shops, fungi, plant trade

## Abstract

Medicinal plants are important in the South African traditional healthcare system, the growth in the consumption has led to increase in trade through *muthi* shops and street vendors. Medicinal plants are prone to contamination with fungi and their mycotoxins. The study investigated multiple mycotoxin contamination using Ultra High Pressure Liquid Chromatography–Tandem Mass Spectrometry (UPLC-ESI-MS/MS) for the simultaneous detection of Aflatoxin B1 (AFB1), Deoxynivalenol (DON), Fumonisins (FB_1_, FB_2_, FB_3_), Nivalenol (NIV), Ochratoxin A (OTA) and Zearalenone (ZEN) in frequently sold medicinal plants. Medicinal plant samples (*n* = 34) were purchased and analyzed for the presence of eight mycotoxins. DON and NIV were not detected in all samples analyzed. Ten out of thirty-four samples tested positive for mycotoxins —AFB_1_ (10.0%); OTA (10.0%); FB1 (30.0%); FB2 (50.0%); FB3 (20.0%); and ZEN (30.0%). Mean concentration levels ranged from AFB_1_ (15 µg/kg), OTA (4 µg/kg), FB_1_ (7–12 µg/kg), FB_2_ (1–18 µg/kg), FB_3_ (1–15 µg/kg) and ZEN (7–183 µg/kg). Multiple mycotoxin contamination was observed in 30% of the positive samples with fumonisins. The concentration of AFB_1_ reported in this study is above the permissible limit for AFB1 (5 µg/kg). Fumonisin concentration did not exceed the limits set for raw maize grain (4000 µg/kg of FB_1_ and FB_2_). ZEN and OTA are not regulated in South Africa. The findings indicate the prevalence of mycotoxin contamination in frequently traded medicinal plants that poses a health risk to consumers. There is therefore a need for routine monitoring of multiple mycotoxin contamination, human exposure assessments using biomarker analysis and establishment of regulations and standards.

## 1. Introduction

There has been a steady increase in the demand for medicinal plants, herbs and preparations as complementary and alternative medicine (CAM) and in traditional medicine both in developing and developed countries [1]. In developed countries, between 25 and 70% of the population rely on complementary and/or alternative medicine (CAM) [2]. In Africa, the use of medicinal plants and consumption is significantly higher, around 80%, due to economic, social, and cultural factors. Medicinal plants play a vital role in disease prevention and their promotion and use compliments current prevention strategies under the Primary Health Care Approach [3,4].

Due to a complex supply chain involving different players and conditions from pre-harvesting, harvesting, storage and trade, medicinal plants are prone to infestation by pests, microbes and toxins [5,6]. Mycotoxins are toxic fungal secondary metabolites and are common contaminants of both human food and animal feed. Contamination is more common in developing countries with poor crop storage and production technologies, and climatic conditions which promote fungal growth and toxin production [7]. There are over 400 mycotoxins known today. Aflatoxins, ochratoxins, fumonisins and trichothecenes are the major classes of mycotoxins that have been recognized as being of public health significance due to their high occurrence and associated carcinogenic properties [6,8].

Mycotoxin exposures occur via various routes of entry such as oral, dermal, respiratory and parenteral. The oral/ingestion route is the major route of entry for mycotoxin exposures. A potential chain reaction can occur when contaminated animal feed results in infected meat, milk and eggs [9] which in turn, can affect human health. Acute and chronic mycotoxicosis can be developed depending on an individual’s susceptibility, the type of mycotoxin and dosage [7,10]. For example, approximately a third of all cases of liver cancer in Africa are due to chronic exposures to mycotoxins [11]. Table 1 shows the adverse effects of some mycotoxins on animal and human health [9,12,13,14,15]. Additive or synergistic harmful effects may also be a result of co-occurring mycotoxins [12]

Despite the reported and potential impacts of mycotoxins including their relation to many diseases, they are poorly studied in South African medicinal plants sold in markets which are prone to contamination [16,17]. Furthermore, the control of mycotoxins is inadequately funded, and many African governments do not give priority to mycotoxin control in medicinal plants [18]. However, the occurrence of mycotoxins has been reported in South Africa [16,17]; Kenya [19,20,21]; Nigeria [22,23,24,25]; and Egypt [26,27,28,29].

Most of these studies have been limited in scope focusing mainly on aflatoxin and fumonisin contamination. In view of the increasing demand for and trade in medicinal plants and the health risks from fungal contamination and their toxins, there is a need to have a broad understanding of the prevalence of mycotoxins in commercially traded medicinal plants. Regrettably, there is limited information on mycotoxins in medicinal plants in South Africa which is not commensurate with the escalating economic value of the trade. As mentioned earlier, previous studies have been completed in South Africa, but no studies have been published on mycotoxin contamination in medicinal plants sold in the Free State Province *muthi* (traditional medicine) shops and by street vendors, hence there is no information available. The aim of this study was to assess the safety of medicinal plants with respect to multiple mycotoxin contamination namely Aflatoxin B_1_ (AFB_1_), Ochratoxin A (OTA), Zearalenone (ZEN), Deoxynivalenol (DON), Nivalenol (NIV) and Fumonisins (FB_1_, FB_2_, FB_3_) as supported by Keter et al. [20].

## 2. Results

### 2.1. Mycotoxin Extraction

Whilst other studies have employed sample clean up, the present study directly injected mycotoxin extracts into the LC-MS/MS without any simple clean up. This is in line with a study on simultaneous LC/MS/MS determination of aflatoxins, fumonisins, OTA and patulin, type A and B trichothecenes and Zearalenone, with no sample clean-up [30] The method employed in this study is also supported by an HPLC-ESI-MS/MS method which was developed for simultaneous determination of 33 mycotoxins in various products. The mycotoxins were extracted with acetonitrile/water and then directly injected into a LC-MS/MS system without any clean-up [31]. Other studies have also conducted mycotoxin analysis with no clean-up step in various matrices [32]. Therefore, the extraction method was quite efficient in isolating the targeted mycotoxins under investigation.

### 2.2. Mycotoxin Analysis

**Figure 1:**
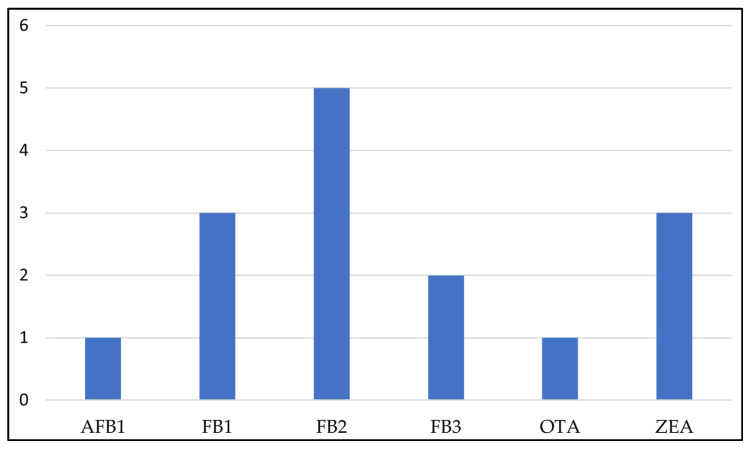
Frequency of occurrence of mycotoxins in medicinal plants.

A total of 34 samples from commonly sold medicinal plants were analyzed for the presence of multiple mycotoxins. None of the plant samples contained detectable levels of DON and NIV. Of the 34 samples, 10 (29%) were positive for OTA (1); AFB_1_ (1); FB_1_(3); FB_2_(5); FB_3_ (2); and ZEN (3) as illustrated in Figure 1. Multi-mycotoxin contamination was observed in 30% of the positive samples with fumonisin derivatives (FB_1_, FB_2_, FB_3_).

The occurrence of mycotoxins and their concentrations in medicinal plants are presented in Table 2. AFB1 was only found in *Dicoma anomala* at a concentration of 15 µg/kg and OTA (4 µg/kg) was found in *Aloe ferox*. FB1 ranged from 1 µg/kg to 12 µg/kg while FB_2_ was detected in five different pants from five different locations. FB_2_ concentrations ranged between 1 µg/kg and 18 µg/kg. FB_3_ (1–15 µg/kg) with a mean of 4.5 µg/kg and ZEN (7–183 µg/kg) with a mean of 81.3 µg/kg. The highest mycotoxin contamination level in the study was recorded for ZEN at 183 µg/kg. A sample contaminated with ZEN had the highest total mycotoxin levels whilst the least contaminated had a concentration of 15 µg/kg.

## 3. Discussion

### 3.1. Aflatoxin (AFB_1_)

Aflatoxins have been reported as mycotoxins of human importance [33]. There are various worldwide reports on aflatoxin contamination in medicinal plants. The current study findings seem to be consistent with a report by Aiko and Mehta, in their study of 63 Indian medicinal herbs samples only one sample tested positive for aflatoxin B [34]. In another study of African traditional herbal medicines sold in South Africa (Tshwane-Pretoria and Cape Town), all 16 samples were not contaminated with aflatoxins [17]. A study in Italy found that all samples of medicinal plants, aromatic herbs and herbal infusions were not contaminated with aflatoxins [29].

Another study of 500 herbal plants in Poland reported all samples to be safe from aflatoxins [35]. This is quite similar to the current study findings which only found one sample to be contaminated with AFB_1_. In contrast, the authors of [36] reported aflatoxin contamination in 58.9% of herbal tea samples from Moroccan market. Tassaneeyakul et al., reported aflatoxin contamination in herbal medicinal plant products in Thailand, in the range of 1.7–0.0000143 µg/kg in 5 out of 28 samples, which is lower than the present findings [37]. The AFB_1_ concentration reported in our study was above the results reported by Yang et al., with AFs (up to 32 µg/kg) in 3 of 19 samples of Chinese herbal medicines [38]. Commonly used Nigerian indigenous crude herbal preparations tested positive for aflatoxin contamination in the range of 0.004–0.345 µg/kg, which is also lower than the current study findings [18]. AFB_1_ has also been reported in kava kava at a concentration of 0.0005 µg/kg. In the same study, other botanical roots’ samples tested negative for aflatoxins [39] In China, the authors of [40] reported one sample of medicinal materials of radix and rhizome to be contaminated with AFB_1_ (5 µg/kg).

The optimum conditions for aflatoxin production by *Aspergillus flavus* and *A. parasiticus* species are at (0.94–0.99 a_w_) and temperatures (25–37 °C) [41,42]. The climate of the Free State province, especially the summer temperatures, might contribute to aflatoxin contamination in medicinal plants whilst another factor might be the climatic conditions where the plants are collected: Gauteng as well as the KwaZulu Natal major markets. The concentrations reported in this study are above the permissible limit for AFB_1_ of 5 µg/kg and total AFs (10 μg/kg) [43]. The AFB_1_ level reported in the study is also above maximum limits of 2 µg/kg for AFB_1_ and 4 µg/kg for total aflatoxins in herbal drugs set by the European Pharmacopoeia [44] as well as Liu et al. [45], who proposed maximum limits of 5 µg/kg and 10 µg/kg for AFB_1_ and total aflatoxins, respectively. The presence of AFB_1_ sheds light on the possibility of contamination of medicinal plants by aflatoxins. Therefore, consumers of medicinal plants sold in the markets might be at risk of mycotoxicosis due to aflatoxin contamination.

### 3.2. Deoxynivalenol (DON) and Nivalenol (NIV)

DON and NIV were not detected in all the samples that were analyzed in the current study. This outcome contrasts with a study conducted in Spain which found 62% of the 84 types of aromatics and/or medicinal herb samples analyzed were contaminated with DON [46]. A study of Chinese medicinal herbs and related products reported DON (17.2–50.5 µg/kg) contamination in 3 out of 58 samples [47]. A most recent study by Darra et al. [48] in Lebanon on multi-mycotoxin occurrence in commercial spices and herbs found DON (12% in spices, 3% in herbs) but NIV was not detected.

In Latvia, DON was detected in 45% of marketed herbal tea samples at concentrations of 129 µg/kg in the herbal blend and 5.463 µg/kg in wormwood tea [49]. Whilst previous studies have detected the presence of DON and NIV, the absence of these mycotoxins may be attributed to the environmental conditions which do not favor the production of these mycotoxins by fungi species. The European Commission has set limits of DON at maximum of 200 µg/kg for processed cereal-based food and 1250 µg/kg for unprocessed cereals [50]. In South Africa, DON is regulated and for maize or barley ready for human consumption they may not contain more than 1000 ug/kg of deoxynivalenol [43]. However, regulatory limits have not yet been provided for NIV.

### 3.3. Fumonisins (FB_1_, FB_2_, FB_3_)

In accordance with the present results, previous studies have demonstrated that medicinal plants can be contaminated with fumonisins. In Turkey, 2% of 115 medicinal plants and herbal tea samples tested positive for FB_1_ at levels of 0.00016 and 0.001487 µg/kg [51]. The current study findings were lower than previous studies in South Africa [16], where it was reported in the Eastern Cape that only 4 out of 30 medicinal wild plants samples tested were contaminated with FB_1_ (8–1553 µg/kg. In another study of African traditional herbal medicines sold in Tshwane and Cape Town, 81% of 16 samples were found contaminated with FB1 (14–139 µg/kg) [17]. These earlier studies concluded that FB_1_ contamination was more common in South African medicinal herbs whilst the current study found FB_2_ to be the predominant fumonisin derivative.

Samples of black tea and medicinal plants sold in Lisbon supermarkets in Portugal 65% tested positive for FB_1_ (range, 20 to 700 μg/kg) whilst none were contaminated with FB_2_ [52]. Han et al. [53] reported that more than 50% of 35 samples of traditional Chinese medicines tested positive for fumonisins’ contamination (0.58–88.95 μg/kg). In a recent study of mycotoxin contamination in Menthae haplocalycis, Luo et al., reported FB_1_ and FB_2_ in the samples analyzed [54]. The presence of fumonisins’ contamination has been reported before which demonstrates the risk to consumers, the need for continuous monitoring and in vitro studies in exposure risk assessments.

In South Africa, fumonisin is regulated for raw maize grain intended for further processing, that may not contain more than 4000 µg/kg of FB_1_ and FB_2_, whereas for maize flour, maize meal ready for human consumption has a limit of 200 µg/kg for FB_1_ and FB_2_ whole commodity [43] The present study findings for total fumonisins were below the regulatory limits, but this does not absolve the consumer from fumonisin-mycotoxin health risks.

### 3.4. Ochratoxin (OTA)

In Poland, 49% of the 79 samples of herbs analyzed for natural occurrence of OTA contamination tested positive whilst 22.3% exceeded OTA acceptable limits [55]. The OTA concentration from this study was higher than the results of Roy and Kumar [56] who reported that 44% of 129 herbal samples destined for Ayurvedic medicines were contaminated with OTA (range 0.3–0.00234 µg/kg). Aziz et al., reported higher OTA levels compared to our findings; in their study, 3 out of 17 medicinal plant samples were contaminated by OTA at a mean concentration of (20–80 µg/kg [25]).

In addition, Bresch et al., reported OTA contamination in 50% of the 19 licorice samples (range, 0.3 to 216 µg/kg) [57]. A study on Chinese medicinal plants reported OTA presence in 44% of the 57 samples analyzed (range 1.2–158.7 µg/kg) [38]. In a recent study Ochratoxin A (OTA) was detected in 10% of herbal teas marketed in Latvia at concentrations that ranged between 2.99–30.3 µg/kg [49].

According to EFSA, OTA has been found in breast milk, which could represent a possible health concern for breast-fed infants [58]. Shim et al., reported an OTA transfer rate of (12.72–61.33%) from herbal medicines to decoctions indicating that the use of mycotoxin-contaminated medicinal plants presents a health risk to the consumers after consumption of such products [59]. OTA is not regulated in South Africa, however, according to the European Union Commission Regulation [50], the maximum residue level (MRL) for OTA in nutmeg, ginger, turmeric, black and white pepper, licorice root and its extract, the legislative limit varies from 15 μg/kg to 80 μg/kg. In the current study, the OTA concentration was below the set limit as well as the European regulatory standard (5 µg/kg in unprocessed cereals) [50].

### 3.5. Zearalenone (ZEN)

The mean concentration for zearalenone recorded in this study was lower than the one reported in China wherein all nine samples of the coix seed medicinal herb tested positive for ZEN (range, 18.7–211.4 µg/kg) [60]. Similarly, another study in China [61] also reported ZEN contamination of coix seeds (68.9 to 119.6 µg/kg). ZEN was not detected in an earlier study of 84 medicinal plant samples using direct determination methods [26]. Different countries have set a maximum limit for ZEN ranging from 20 to 1000 µg/kg in raw and processed food items [50]. In our study, ZEN contamination levels (7–183 µg/kg) did not exceed the permissible limits. ZEN-advanced pubertal changes in young children have been reported in Puerto Rico and gynecomastia with testicular atrophy has been reported in rural males in Southern Africa [62,63].

### 3.6. Multiple Mycotoxin Contamination

In this study, the mycotoxin occurrence was mainly extracted from the fumonisin derivatives (FB_1_, FB_2_, FB_3_). Mycotoxin co-occurrence has been reported in previous studies of medicinal plants. In Spain, all 84 samples of medicinal and aromatic herbs analyzed showed multi-contamination with AFs, OTA, ZEN, FBs, DON, T-2 toxin and citrinin [46]. Another study reported contamination in 20.58% of the powdered herbal samples with mycotoxins (total aflatoxins, sterigmatocystin, citrinin) [64]. In an analysis of ginger products, aflatoxins and OTA were detected in 67% and 74% samples, respectively, with a range of 0.001–0.03 ng/kg [65]. A study by Koul and Sumbali, [66], found the presence of ZEN and DON in 13.07% and 6.92% of 130 samples of medicinally important dried rhizomes and root tubers. Veprikova et al., analyzed herbal-based dietary supplements for the presence of 57 mycotoxins. The study reported Fusarium trichothecenes, ZEN and ENs and Alternaria as the main mycotoxins and mycotoxin co-occurrence of ENs, HT-2, T-2 and Alternaria toxins [67].

A simultaneous analysis of multiple mycotoxins in 44 samples of *Alpinia oxyphylla* by UPLC-MS/MS detected AFB_1_, ZEN, OTA, FB_1_ and FB_2_ in four moldy samples [68]. Another study of multiclass mycotoxins in Chinese medicinal and edible lotus seeds found three of the ten batches of samples tested positive for AFB_1_, FB_2_, T-2 and ZEN [69]. An investigation into the presence of multi-class mycotoxins in 40 batches of *Menthae haplocalycis* samples found the most common mycotoxin was tentoxin, followed by alternariol, alternariol monomethyl ether, ZEN, FB_2_, FB_3_, OTA, AFB_1_, AFB_2_, AFG_1_ and T-2 toxin [54].

Reinholds et al. [49] analyzed 60 samples of herbal teas from Latvia drugstores for the presence of 12 mycotoxins. Among the dry tea samples, 90% were positive for at least one–eight mycotoxins. A study on teas and medicinal plants used to prepare infusions in Portugal reported that 84% of the analyzed samples tested positive for at least one of the mycotoxins [70]. Narvaez, in the analysis of the presence of 16 mycotoxins in botanical nutraceuticals, reported a co-occurrence in 4 out of 10 samples (EN B1, EN A and EN A1). Meanwhile, the prevalent mycotoxins were ZEN (60%) and EN B1 (30%) in samples analyzed [71]. A recent study by Caldeirão et al. [72], analyzed 58 herbs from Brazil for the presence of 14 mycotoxins by LC-MS/MS. Mycotoxin multiple contamination (range 1–8) was reported in 72% of the samples. The most prevalent mycotoxins were enniatins (EN), beauvericin (BEA), sterigmatocystin (STE) and HT-2 toxin, whilst FB_1_, FB_2_, and T-2 were not detected in any of the samples. Furthermore, the concentration of mycotoxins in the herbal infusions was 88% lower than in the raw herbs.

The percentage of positive samples and mycotoxin co-occurrence of mycotoxins varied among the different studies. This can be attributed to the sensitivity of the methods used and the wide range of mycotoxins analyzed as compared to the current study which only investigated the presence of eight mycotoxins. The reports from preceding studies further indicate that multiple mycotoxin contamination in medicinal plants, herbs and herbal products is cause of concern. Therefore, there is need for a comprehensive analysis of other emerging mycotoxins in routine monitoring of medicinal plants and their products.

The presence of mycotoxins in human food and animal feed increases the risk of endemic diseases such as malaria, hepatitis and HIV with consequent acute and chronic effects [8]. The lack of epidemiological studies, focusing on co-exposure to multi-class mycotoxins and associated health outcomes, is partly attributable to the absence of valid biomarkers [8,73]. A study of 53 South African women, found eight single or combined mycotoxins in urine samples including: DON; FB_1_; OTA; and ZEN [73]. In another study conducted in Cameroon, the authors reported the detection of 11 single or combined mycotoxins and their metabolites in 63% of 175 urine samples including AFM_1_, OTA and DON [74]. The presence of more than one mycotoxin demonstrates the possibility of mycotoxin exposures from single or multiple sources not limited to food but also from other non-food sources as reported earlier. Therefore, the contribution of medicinal plants as source of mycotoxin exposures should not be underestimated. Whilst co-occurrence has been reported in medicinal plants by several previous studies, present study findings warrant further research to analyze for a wide range of mycotoxins, especially the ones not frequently studied/reported.

## 4. Conclusions

The study evaluated the presence of mycotoxins (AFB_1_, DON, FBs, NIV, OTA, and ZEN) as they have been reported to be the major mycotoxins of public health importance. The findings indicate the prevalence of mycotoxin contamination in frequently traded medicinal plants in South Africa. Mycotoxins pose a health risk to consumers due to the additive or synergistic effects of mycotoxins. Taking into consideration the frequency of use, dietary intakes and individual susceptibility among other factors, consumers are at an increased risk from mycotoxins and their adverse health effects. The current study’s findings, supported by previous urinary biomarkers’ assessment reports, demonstrate that consumers of medicinal plants are at risk despite the low concentration levels recorded for some mycotoxins in medicinal plants and their products. This is the first study in the Free State Province, South Africa to investigate multiple mycotoxin contamination in marketed medicinal plants. There is therefore a need for routine monitoring of multiple mycotoxins and regulations as well as human exposure assessments using biomarker analysis. Inspections of storage and trading conditions, including regulation of trade, are required to ensure that the trade in medicinal plants is conducted in environments that do not favor the growth of fungi and mycotoxin production.

## 5. Materials and Methods

### 5.1. Standards and Reagents

The mycotoxin standards comprising of Aflatoxin B_1_ (AFB_1_), Zearalenone (ZEN), Nivalenol (NIV), Deoxynivalenol (DON) and Ochratoxin A (OTA), were obtained from Sigma-Aldrich (Bornem, Belgium). Fumonisin (B_1_, B_2_, B_3_) was purchased from Promec Unit (Tynberg, South Africa). Acetonitrile (VWR International, Zaventem, Belgium) and methanol (Biosolve, Valkenswaard, The Netherlands), Formic acid (≥98%) (Merck, Darmstadt, Germany). All reagents were of analytical grade.

### 5.2. Sample Collection and Preparation

A survey was carried out throughout the Free State province, South Africa where 48 vendors were asked to list their top ten selling medicinal plants. Participants listed 165 medicinal plants. The plants which had a Frequency Index (percentage frequency of mention for a single species by informants) ≥ 10 were selected for further analysis. A total of 34 samples from 32 plant species (Table S1) were randomly selected and purchased from *muthi* shops and street vendors. The samples that were procured from the *muthi* shops (16) and street vendors (18) comprised of roots, bark, leaves, stems and bulbs. Samples were collected in a dry state in sterile zip-lock plastic bags and immediately transported to the CAFSaB laboratory at the Central University of Technology. Samples were further dried to reduce moisture content in a laminar air flow dryer (Lasec). All dried samples were milled using a Kinematica Polymix PX-MFC90D (Kinematica AG, Luzern, Switzerland) to less than 0.5 mm particle size. Homogenized samples of 30 g were divided into two, for mycotoxin and microbial analysis. Samples were stored in sterile zip-lock bags at 4 °C to inhibit mycotoxin production and fungal growth until analysis.

### 5.3. Mycotoxin Extraction

A simple solvent extraction method with no sample clean-up was used as described by Spanjer [27]. Homogenized samples were accurately weighed (approx. 5 g) using an analytical balance (3 dp) into a 50 mL tube. Extraction solvent of 20 mL of water/methanol/acetonitrile (2/1/1, *v*/*v*) was added and sonicated for 60 min. Then, 1 mL of sample was aliquoted into a 2 mL Eppendorf tube and double diluted with 75% water; 25% methanol solvent and centrifuged for 5 min at 13,000 rpm. A total of 1 mL of the diluted sample was aliquoted into an analysis vial for analysis.

### 5.4. Equipment Calibration

A calibration graph was created by plotting the obtained peak area or peak height for each standard working solution against the mass of each mycotoxin injected. Each mycotoxin peak in the chromatogram was identified by comparing the retention times with those of corresponding reference standards. The quantity of mycotoxins in injected eluate was determined by comparison to the respective standard curves of each mycotoxin standard.

### 5.5. Liquid Chromatography Tandem Mass Spectrometry

A Waters Acquity Ultra Performance Liquid Chromatography (UPLC) apparatus coupled to a Xevo Triple Quadrupole Tandem Mass Spectrometer (TQMS) (Waters, Milford, MA, USA) was used for high resolution UPLC/MS/MS for the detection and quantification of mycotoxins. A symmetry Waters column UPLC BEH -C18 (100 mm × 2.1 id; 1.7 µm particle size) attached to a guard column (10 mm × 2.1 mm i.d.) (Waters, Zellik, Belgium) was used. A fixed sample injection volume of 2 µL was used. Mobile phase solvent A consisted of acidified water with 0.1% formic acid (10/1, *v*/*v*) and mobile phase solvent B of 0.1% formic acid in acetonitrile acidified with 0.1% formic acid (10/1, *v*/*v*). Multiple mycotoxins were separated in the mass spectrometer operated using selected multiple reaction monitoring channels (MRM) in positive electrospray ionization mode (ESI+). ESI conditions were optimized as follows; capillary voltage, 3.5 V; cone voltage range, 15–50 V; collision energy range, 10–40 eV; source temperature, 140 °C. Nitrogen was used as the desolvation gas, desolvation temperature of 400 °C; desolvation gas, 800 L/h and cone gas, 50 L/h.

The gradient elution program (illustrated in Figure S1) at initial conditions of 98% A (Water + 0.1% formic acid), held for 0–0.5 min at a flow rate of 0.35 mL/min was used. This was followed by a slow gradient change of solvent A to 60% from 0.5–7 min. From 7–10 min there was another gradient change of solvent A to 30%. A rapid gradient change ensued for solvent A to 5% from 10–11 min. There was another gradient change of solvent A to 0% from 11–12 min. This was followed by a quick gradient change to initial conditions of 98% solvent A from 12–12.1 min. After that, an isocratic period of 98% of solvent A was kept for 12.1–14 min. The column was reconditioned with solvent B (Acetonitrile + 0.1% formic acid) for 5 min before the next injection. The total analytical run time was 14 min through a linear decrease of mobile phase.

### 5.6. Data Acquisition and Analysis

The frequency index (FI) used to select the plants used in this study was calculated using the formula FI = (FC ÷ N) × 100. FC is the number of informants who mentioned the use of the species, and N is the total number of informants. N = 48 in this study. Plant names were documented in the local languages, mostly Sotho and Zulu, and scientific names identified from literature.

MassLynx and QuanLynx software’s version 4.1 (Micromass, Manchester, UK) were used for data acquisition and processing. Descriptive statistics (mean, range, maximum and the frequency of the data obtained in this study) were calculated using Microsoft Office Excel 2016.

## Figures and Tables

**Table 1:** Mycotoxigenic effects on animal and human health.

Mycotoxin	Fungal Source (Genus)	Health Effects
Aflatoxin B1	*Aspergillus*	Teratogenic, hepatotoxic, immunosuppressive, carcinogenic and mutagenic.
Deoxynivalenol	*Fusarium*	Gastrointestinal damage, reproductive effects toxicosis, genotoxicity and immunosuppressive.
Fumonisins	*Fusarium*	Teratogenic, carcinogenic, hepatotoxic, nephrotoxic, immunosuppressive and neurotoxic.
Nivalenol	*Fusarium*	Anorexic, immunotoxic, hematotoxic and genotoxic.
Ochratoxin A	*Aspergillus* *Penicillium*	Carcinogenic, teratogenic, immunosuppressive and nephrotoxic.
Zearalenone	*Fusarium*	Carcinogenic, hormonal imbalance (hyperestrogenism) and reproductive effects.

**Table 2:** Mycotoxin contamination in medicinal plants.

Plant Name	Trader: Location	AFB_1_	FB_1_	FB_2_	FB_3_	OTA	ZEN
*Bulbine narcissifolia* Salm-Dyck	MS: Thaba ‘Nchu	-	10.0	18.0	1.0	-	-
*Helichrysum odoratissimum* (L.) Sweet.	SV: Zastron	-	12.0	15.0	-	-	-
*Hypoxis hemerocallidea* Fisch., C.A.Mey. & Avé-Lall.	MS: Dewetsdorp	-	-	-	-	-	183.0
*Adenia gummifera* (Harv.) Harms	MS: Sasolburg	-	-	-	-	-	54.0
*Aloe ferox* Mill.	MS: Senekal	-	-	-	-	4.0	-
*Galium capense* Thunb	MS: Winburg	-	-	2.0	-	-	-
*Siphonochilus aethiopicus* (Schweif.) B.L. Burt	SV: Kroonstad	-	-	-	-	-	7.0
*Helichrysum odoratissimum* (L.) Sweet.	SV: Kroonstad	-	-	6.0	-	-	-
*Dicoma anomala* Sond.	SV: Bloemfontein	15.0	-	-	-		
*Pentanisia prunelloides* (Klotzsch ex Eckl. & Zeyh.) Walp.	SV: Bloemfontein	-	7.0	1.0	1.0	-	-
Mean of positive samples ± standard deviation		15.0	9.6 ± 2.5	8.4 ± 7.7	1.0	4.0	81.3 ± 91.1

Only positive sample results have been shown; Concentrations in µg/kg; Not detected (-). SV-Street Vendor; MS-*Muthi* Shop.

## Data Availability

Not applicable.

## Supplementary Materials

The following supporting information can be downloaded at: https://www.mdpi.com/article/10.3390/toxins14100690/s1, Figure S1: Gradient elution program; Table S1: Medicinal plants screened for mycotoxins.
