# Supplementary material for: Multiple Mycotoxin Contamination in Medicinal Plants Frequently Sold in the Free State Province, South Africa Detected Using UPLC-ESI-MS/MS
Source: Toxins (Basel). 2022 Oct 8;14(10):690. doi: 10.3390/toxins14100690 (PMC9607566; doi:10.3390/toxins14100690)
Supplement: Supplementary file 1 [file toxins-14-00690-s001.zip › toxins-1882961-supplementary.pdf]

Supplementary Materials: Multiple Mycotoxin Contamination in Medicinal Plants Frequently Sold in the Free State Province, South Africa Detected Using UPLC-ESI-MS/MS

Julius Ndoro, Idah Tichaidza Manduna, Makomborero Nyoni and Olga de Smidt

Table S1. Medicinal plants screened for mycotoxins.

Scientific Name	Family	Local Name	FI (%)	Sample	Location
Hypoxis hemerocallidea Fisch., C.A.Mey. & Avé-Lall.	Hypoxidaceae	Ilabatheka / Moli	38	MS11	Dewetsdorp
Dicoma anomala Sond.	Asteraceae	Hloenya	33	SV07	Zastron
Elephantorrhiza elephantina Burch. Skeels	Fabaceae	Mosetsane	31	SV10	Bethlem
Helichrysum odoratissimum L. Sweet	Asteraceae	Mpepa	31	SV14	Kroonstad
Tulbaghia alliacea L.f.	Alliaceae	Umwelela / Molela	25	MS10	Dewetsdorp
Pentanisia prunelloides Klotzsch ex Eckl. & Zeyh.	Rubiaceae	Setima-mollo	23	SV02	Bloemfontein
Bulbine narcissifolia Salm-Dyck	Asphodelaceae	Kgomo-ea - balisa	19	MS13	Thaba Nchu
Alepidea amatymbica Eckl. & Zeyh	Apiaceae	Lesoko	19	MS15 & SV19	Welkom & Bethlehem
Xysmalobium undulatum L.	Apocynaceae	Poho-tshehla/Pohotshehele	19	SV04	Thaba 'Nchu
Clivia miniata (Lindl.) Regel	Amaryllidaceae	Umayime	17	MS05	Sasolburg
Eucomis autumnalis Mill. Chitt.	Asparagaceae	Umathunga	17	MS07	Qwaqwa
Aloe ferox Mill.	Asphodelaceae	Khala	15	MS04	Senekal
Drimia depressa Baker Jessop	Hyacinthaceae	Moretele	15	MS16	Sasolburg
Talinum caffrum Thunb	Portulacaceae	Punyuka bamphethe/ khutsana	13	MS02	Parys
Euclea coriacea A.DC.	Ebenaceae	Monna-mots'o	13	SV08 & SV18	Qwaqwa
Dianthus basuticus Burtt Davy	Caryophyllaceae	Hlokoa-la-tsela	13	SV16	Kroonstad
Pappea capensis Eckl. & Zeyh	Sapindaceae	Voma	11	SV15	Welkom
*Adenia gumm*ifera Harv. Harms	Passifloraceae	Impinda	10	MS06	Sasolburg
Cussonia paniculata Eckl. & Zeyh	Araliaceae	Moretseng/Mots'ets'e	10	SV11	Senekal
Helichrysum caespititium DC	Asteraceae	Phate-ea-ngaka	10	SV12	Parys
Podocarpus henkelii Stapf ex Dallim. & Jacks	Podocarpaceae	Vhulakhuvhaliwe	10	SV17	Kroonstad
Galium capense Thunb	*Rubiaceae*	Mabona	10	MS03	Winbrug
*Crotalaria nata*lia Meisn	Fabaceae	Uvelabahleke	10	MS08	Ladybrand
Scabiosa columbaria L.	Dipsacaceae	Selomi	10	MS09	Ladybrand

Hermannia depressa N.E.Br.	Malvaceae	Seletjane	10	MS12	Bultfontein
Leucosidea sericea Eckl. & Zeyh.	Rosaceae	Cheche	10	MS14	Bloemfontein
Sclerochiton ilicifolius A.Meeuse	Acanthaceae	Molomo monate	10	SV01	Welkom
Curtisia dentata Burm.f. C.A.Sm.	Cornaceae	Tshwene	10	SV09	Bethlem
Siphonochilus aethiopicus Schweif. B.L. Burt	Zingiberaceae	Isiphepheto	10	SV13	Kroonstad

FI–Frequency Index; SV–Street Vendor; MS–*Muthi* Shop

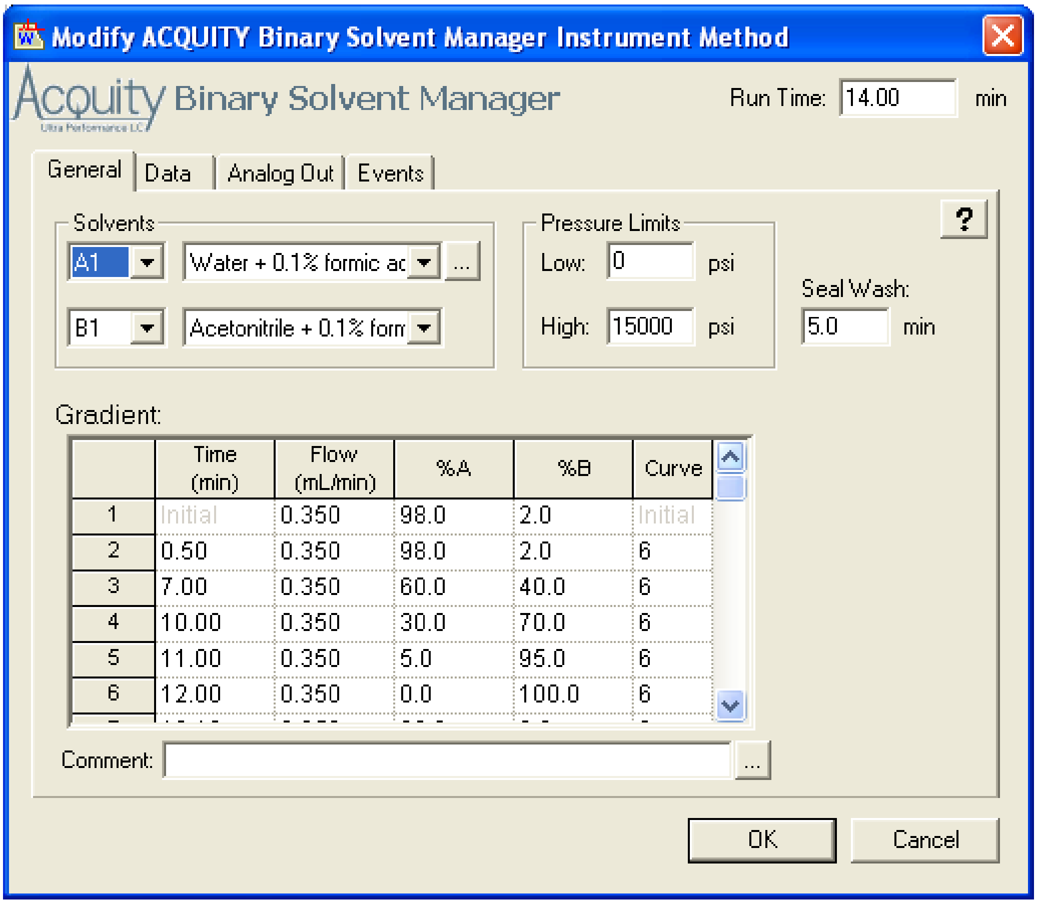

	Time (min)	Flow (mL/min)	%A	%B	Curve
1	Initial	0.350	98.0	2.0	Initial
2	0.50	0.350	98.0	2.0	6
3	7.00	0.350	60.0	40.0	6
4	10.00	0.350	30.0	70.0	6
5	11.00	0.350	5.0	95.0	6
6	12.00	0.350	0.0	100.0	6

Figure S1. Gradient elution program for Liquid Chromatography Tandem Mass Spectrometry.
